# Impact of pre-existing MSP1_42_-allele specific immunity on potency of an erythrocytic *Plasmodium falciparum* vaccine

**DOI:** 10.1186/1475-2875-11-315

**Published:** 2012-09-07

**Authors:** Elke S Bergmann-Leitner, Elizabeth H Duncan, Ryan M Mease, Evelina Angov

**Affiliations:** 1 Malaria Vaccine Branch, US Military Malaria Vaccine Program, Walter Reed Army Institute of Research, 503 Robert Grant Ave, Silver Spring, MD 20910, USA

**Keywords:** Pre-existing immunity, *Plasmodium*, Vaccine, Erythrocytic stage, Merozoite surface protein-1, Clonal imprinting

## Abstract

**Background:**

MSP1 is the major surface protein on merozoites and a prime candidate for a blood stage malaria vaccine. Preclinical and seroepidemiological studies have implicated antibodies to MSP1 in protection against blood stage parasitaemia and/or reduced parasite densities, respectively. Malaria endemic areas have multiple strains of *Plasmodium falciparum* circulating at any given time, giving rise to complex immune responses, an issue which is generally not addressed in clinical trials conducted in non-endemic areas. A lack of understanding of the effect of pre-existing immunity to heterologous parasite strains may significantly contribute to vaccine failure in the field. The purpose of this study was to model the effect of pre-existing immunity to MSP1_42_ on the immunogenicity of blood-stage malaria vaccines based on alternative MSP1 alleles.

**Methods:**

Inbred and outbred mice were immunized with various recombinant *P. falciparum* MSP1_42_ proteins that represent the two major alleles of MSP1_42_, MAD20 (3D7) and Wellcome (K1, FVO). Humoral immune responses were analysed by ELISA and Luminex^TM^, and functional activity of induced MSP1_42_-specific antibodies was assessed by growth inhibition assays. T-cell responses were characterized using *ex vivo* ELISpot assays.

**Results:**

Analysis of the immune responses induced by various immunization regimens demonstrated a strong allele-specific response at the T cell level in both inbred and outbred mice. The success of heterologous regimens depended on the degree of homology of the N-terminal p33 portion of the MSP1_42_, likely due to the fact that most T cell epitopes reside in this part of the molecule. Analysis of humoral immune responses revealed a marked cross-reactivity between the alleles. Functional analyses showed that some of the heterologous regimens induced antibodies with improved growth inhibitory activities.

**Conclusion:**

The development of a more broadly efficacious MSP1 based vaccine may be hindered by clonally imprinted p33 responses mainly restricted at the T cell level. In this study, the homology of the p33 sequence between the clonally imprinted response and the vaccine allele determines the magnitude of vaccine induced responses.

## Background

**Figure 1:**
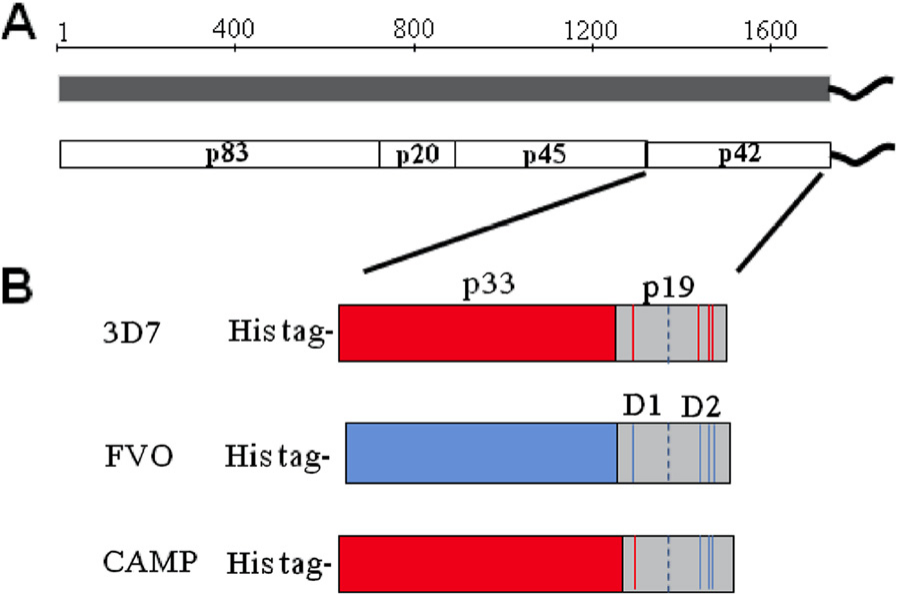
**(A) Schematic diagram of the peptide organization of the MSP1 protein.** (**B**) Schematic of recombinant *E. coli* expressed MSP1_42_ alleles (3D7 = MAD20 (red), FVO = Wellcome/K1 (blue) and the CAMP/FUP parasite clones.

Natural immunity against malaria is based on the presence of antibodies directed against the blood stage parasite, as demonstrated by passive transfer experiments of immunoglobulins [1-3]. There are likely many target antigens that mediate this immunity; however, their identification is obscured by the presence of malaria-specific antibodies that do not necessarily correlate with an efficacious immune response. Characterization of immune complexes formed by merozoites and antibodies from malaria-exposed individuals [4], and results from seroepidemiological studies support the development of the major merozoite surface protein-1 (MSP1) as a vaccine candidate [5-7]. The initial 195kD MSP1 protein undergoes two successive proteolytic cleavage events [8]; the first yielding a non-covalently associated complex formed by the p83, p20 and p45 fragments and the membrane-anchored p42 (see schematic Figure 1A). The second processing event occurs immediately before invasion, resulting in the cleavage of the p42 molecule into a p33 and a p19 fragment. The p19 fragment remains attached to the merozoite surface through a glycosylphosphatidylinositol (GPI) anchor [9] and is comprised of two epidermal growth factor (EGF)-like domains [10], which may play a role in the invasion process. Antibodies directed to the C-terminal fragments of MSP1 (MSP1_19_ and MSP1_42_) have been associated with immunity in preclinical models [11-13].

In the course of characterizing immune responses induced by MSP1 vaccines, it was recognized that: (1) proteins produced by various expression systems differ in their immunogenicity and ability to induce anti-parasite activities [11,13-15]; (2) not all MSP1-based vaccines induce protective immunity in preclinical models [11,13,16,17]; (3) the immunity induced by MSP1_42_ vaccines in nonhuman primate models is parasite strain-specific [13,18]; (4) the degree of parasite inhibition by immune serum induced with an MSP1_42_ vaccine depends on the method chosen to measure invasion- and growth inhibition [19]; and (5) the immunogenicity induced by vaccination with MSP1_42_ and AMA-1 vaccines depends on the malaria exposure history of the vaccinees, *i.e.,* differences in the magnitude of the humoral immune response between US malaria-naïve and African malaria-exposed vaccinees [20-24]. The observed strain specificity arises from the dimorphic nature of MSP1_42_ represented by the two major allelic families, the MAD20 and the Wellcome/K1 [25,26]. At the amino acid level, these two alleles of MSP1_42_ differ by only four amino acids in their p19 region (E-TSR and Q-KNG, respectively), while they differ significantly in their p33 regions exhibiting only 46% identity. Previous studies have mapped dominant T cell epitopes within the p33 region; these epitopes provide help for the humoral response to the highly conserved and disulfide-constrained C-terminal p19 [27-29]. The sequence heterogeneity found in the variant T cell epitopes from different alleles inhibit T cell memory functions and likely interfere with B cell help [18,30]. Thus, inclusion of polymorphic p33 alleles may be required to broadly enhance MSP1 immunogenicity and thus vaccine efficacy.

The purpose of the current study was to simulate pre-existing immunity in mice by establishing primary immune responses with different alleles of MSP1_42_ and to identify whether the established immunity against one allele interferes with the induction of an immune response against the alternative allele. The limited clinical efficacy of MSP1 vaccines in the field, despite good immunogenicity in malaria-naïve US subjects, has led us to formulate the hypothesis that pre-exposure of vaccinees to natural infection interferes with the induction of protective immunity. The concept of original antigenic sin (*i.e.*, exposure to a certain serotype of a pathogen that modulates the induction and magnitude of subsequent immune responses to cross-reactive serotypes and/or pathogens) was first described for the influenza virus [31], but has since been found to apply to other pathogens as well (reviewed in [32,33]). These findings would have implications not only for the broadening of immunity induced by vaccination but also for boosting by natural exposure, and would render an individual more susceptible to infection with alleles not previously encountered.

The previously developed MSP1_42_ vaccines [11,34] represent the two major parasite clones of MSP1_42_, 3D7 (MAD20) and FVO (Wellcome/K1). To address the effect of pre-existing immunity, immunization regimens were designed that employ different prime and boost combinations of MSP1_42_ proteins, namely 3D7, FVO and CAMP/FUP. The third clone, CAMP/FUP, is a hybrid between 3D7 and FVO at the level of p42 and was prevalent in Western Kenya [35] where several clinical trials were conducted evaluating the MSP1_42_ 3D7 vaccine (FMP1/AS02_A_) [20-22,24]. Systematic deep sequencing to identify circulating variants of p19 revealed that the three allotypes of MSP1_42_ were represented at different levels at this site, *i.e.* CAMP/FUP at 58%, FVO at 31% and 3D7 at 7% (C.F. Ockenhouse unpublished observation). For the current study, inbred BALB/c and outbred ICR mice were immunized to determine whether any of the responses are subject to genetic restriction. Humoral immune responses were first characterized by fragment-specific ELISA and flow-based bead assays (Luminex™) and then tested for anti-parasite activity by a parasite lactate dehydrogenase (pLDH) based growth inhibition assay (GIA). Cellular immune responses and the changes in the precursor frequency of MSP1-specific T cells were measured by IFN-γ and IL-4 specific ELISpot assays. Humoral responses were overall cross-reactive with the other alleles, but the highest antibody concentrations were measured against the homologous p42 proteins. The MSP1_42_ CAMP allele was inferior in priming immune responses that could be boosted by the other MSP1_42_ alleles. Priming with either the 3D7 or FVO vaccine followed by a boost with any of the other antigens was always associated with a boosting effect. The best combinations for inducing broadly cross-reactive responses were *ad hoc* mixtures of the 3D7 and FVO antigens (3D7/FVO mix) for both prime and boost, and the heterologous regimen, 3D7 → CAMP or FVO → CAMP (*i.e*. prime → boost). Cellular immune responses were strain-specific as demonstrated by the magnitude of the responses to the homologous proteins. Combinations of 3D7 and FVO (3D7/FVO mix) or using a heterologous prime → boost regimen (3D7 → FVO or FVO → 3D7) led to broadened IFN-γ responses against the p42 FVO and p42 3D7. Notably, CAMP induced weak cross-reactive responses against the other stimulating antigens in a heterologous regimen.

In conclusion, the current study provides evidence that pre-existing allele-specific immunity (acquired immunity) does affect the ability of an MSP1_42_-based protein vaccine to mount an immune response (vaccine immunity). The homology of the p33 subunit between the allele that primed the immune response and the allele used for vaccination will determine whether the established (pre-existing) immune response will be boosted or a new T cell response will be mounted against the “vaccination” allele.

## Methods

### Antigens

*Escherichia coli*-expressed recombinant MSP_42_ 3D7 (FMP1, falciparum malaria protein 1) and codon-harmonized MSP1_42_ FVO (FMP010) were produced under cGMP conditions as previously described [11,34,36]. The recombinant MSP1_42_ CAMP protein was similarly expressed and purified to homogeneity, however, not under cGMP conditions [37]. Subunit fragments of MSP1_42_ (for 3D7 p19, EGF-like domain 1 and EGF-like domain 2; for FVO, p33, p19 and EFG-like domain 1 and EGF-like domain 2) were expressed as fusions to GST and were purified to homogeneity using Glutathione Sepharose 4B resin (GE Healthcare, Piscataway, NJ) [11]. Recombinant GST was purified under the same conditions and used as a negative control in the Luminex assay and for ELISpot. MSP1_33_ 3D7 recombinant protein contained an N-terminal histidine tag and was purified to homogeneity using Ni^+2^-NTA Superflow resin (Qiagen Chatsworth, CA).

### Immunizations

BALB/c mice (5–6 week old, Jackson Laboratories, Bar Harbor ME) were immunized subcutaneously with 2 μg of recombinant *P. falciparum* MSP1_42_ proteins (in the case of 3D7/FVO vaccine mix 1 μg of each antigen was combined). All doses were in 100 μL on day 0 and 21. ICR mice (5–6 week-old, Charles River Laboratories, Wilmington, MA) were immunized with 5 μg of recombinant *P. falciparum* MSP1_42_ proteins (in the case of 3D7/FVO vaccine mix 2.5 μg of each antigen was combined), adjuvanted with Montanide ISA-720 (Seppic Inc., Fairfield, NJ) at three week intervals. Emulsification of the antigen-adjuvant mixture was performed as described elsewhere [38]. Quality control for the Montanide ISA-720 oil emulsions were performed by microscopically examining the size, homogeneity and number of oil droplets in the formulation as per manufacturer’s instructions (Seppic Inc.). Mice were injected in the inguinal area within 1 hr of preparing the emulsion (n = 20/group). Blood samples were obtained two weeks after each immunization.

### ELISA

ELISAs were performed as previously described in detail [39]. Briefly, 96-well plates (Immunolon 2 HB, Thermo, Milford, MA) were coated by overnight incubation with the recombinant MSP1_42_ at 0.8 pmol in 100 μL (0.35 ng/μL) PBS at 4°C. Plates were washed with PBS/0.1% Tween 20 using a 96-well plate automatic ELISA-plate washer (Skatron, Sterling, VA) and blocked with blocking buffer (PBS, 1% BSA, pH 7.4) for 1 hr at 37°C. Sera were diluted in blocking buffer and incubated for 2 hr at 37°C. Alkaline phosphatase-conjugated goat anti-mouse IgG (Promega, Madison, WI) secondary antibody was added to blocking buffer (1:1,000) and incubated for 1 hr at RT. The assay was developed for 15 min at 37°C with BluePhos substrate (Kirkegaard Perry, Gaithersburg, MD) and read at 570 nm. Antibody concentration was determined by establishing a standard curve with purified mouse IgG.

### ELISpot

ELISpot assays were performed as previously described in detail [39]. Briefly, multiscreen plates (Millipore, Bedford MA) were coated with either anti-IFN-γ or anti-IL-4 capturing antibodies according to manufacturer’s instructions (R&D Systems, Minneapolis, MN). Plates were blocked using culture medium (DMEM containing 10% FBS containing Pen/Strep, HEPES, NEAA, sodium pyruvate, 2-mercaptoethanol). Thawed splenocytes were counted and plated at 10^7^ cells/mL (50 μL/well) and stimulated with the various recombinant proteins. Plates were incubated for 36 hrs (IFN-γ) or 48 hrs (IL-4) in the presence of antigen at 37°C and then processed according to manufacturer’s instructions. Plates were counted using the AID Autoimmun Diagnostica GmbH ELISpot reader and software (Strassberg, Germany).

### Luminex™ analysis

Antibody fine specificity to the fragments of MSP1_42_ (p33, p19, EGF-like Domain 1 (D1) and EGF-like Domain 2 (D2)) was detected by the particle-based Luminex™ method. Beads were coupled with 25 ng/5,000 microspheres/well per antigen. Microfilter plates were blocked with 200 μL/well of PBS, 0.05% Tween 20, followed by 200 μL PBS, 1% BSA for 1 hr at RT. 5,000 microspheres of each fragment per 50 μL were combined in the following mixtures to avoid competition or interferences: Multiplex 1: p33 + p19 3D7; Multiplex 2: pD1 + pD2 3D7; Multiplex 3: p33 + p19 FVO; Multiplex 4: pD1 + pD2 FVO. Each reaction contained 50 μL of the microsphere mix with 50 μL of the post boost-diluted serum tested at either 1:200 or 1:2,500. Plates were incubated for 1 hr at 22°C on a vibrating plate shaker (Heidolph Titramax 100). R-phycoerythrin conjugated anti-mouse IgG (1:250) (Jackson ImmunoResearch Laboratories, Inc., West Grove, PA) was added to washed plates (4 times with 200 μL 1X PBS, 0.05% Tween 20) and incubated for 30 min at 22°C on a plate shaker. All data were analysed on a Luminex™ 200 (Luminex 100 IS 2.3 software) set to read the fluorescence intensity per microsphere signature, 100 events per signature (per antigen) were calculated and reported as the mean fluorescence intensity (MFI).

### Parasite cultures and growth inhibition assays

Complete media was prepared with RPMI 1640 (Invitrogen, Carlsbad, CA) containing 25 mM HEPES, 7.5% w/v NaHCO_3_ and 10% human pooled serum (blood group O+). *Plasmodium falciparum* clones 3D7 and FVO were maintained and synchronized by the temperature cycling method [40]. Unless stated otherwise, cultures in the presence or absence of immune serum were set up at approximately six hours before rupture occurred (starting parasitaemia at 0.3%, hematocrit of uninfected erythrocytes at 2%) in 384-well plates under static conditions.

### Immunoglobulin purifications

Mouse IgG from sera were purified using caprylic acid/ammonium sulfate and dialyzed as previously described [41]. Immunoglobulins were pre-absorbed with human red blood cells (RBCs; blood group O+) and tested for GIA by measuring the inhibition of pLDH activity as previously described in detail [42].

### Statistical analysis

Serological responses measured by ELISA, Luminex^TM^ and for GIA were tested for statistical significance using an ANOVA (pairwise comparisons were done by two-sided T-tests) and cellular responses measured by ELISpot were tested by using two-sided T-tests employing the Minitab software package (Penn State University, State College, PA) and Sigmaplot 11.0 (Systat Software Inc). Antibody data tested by ANOVA were equally varied and normally distributed; therefore, no transformation was required.

## Results

### Homologous and heterologous immunization regimens to simulate pre-existing immunity to different alleles of MSP1_42_

Various immunization regimens were designed employing the C-terminal MSP1_42_ of the full length MSP1 from three laboratory parasite isolates (Figure 1), namely 3D7, FVO and CAMP. While 3D7 and FVO represent the two dimorphic alleles of the p33 protein (MAD20 and Wellcome/K1, respectively) the CAMP haplotype represents a hybrid between these two parasite clones where the p33 and EGF-like D1 portion of the molecule is homologous to p42 3D7 and the p19 EGF-like D2 portion is identical to EGF-like D2 of p42 FVO (see Figure 1B).

### Serological responses of inbred and outbred mice to the various vaccine alleles differ significantly but confirm the value of immunizations with mixed alleles

**Figure 2:**
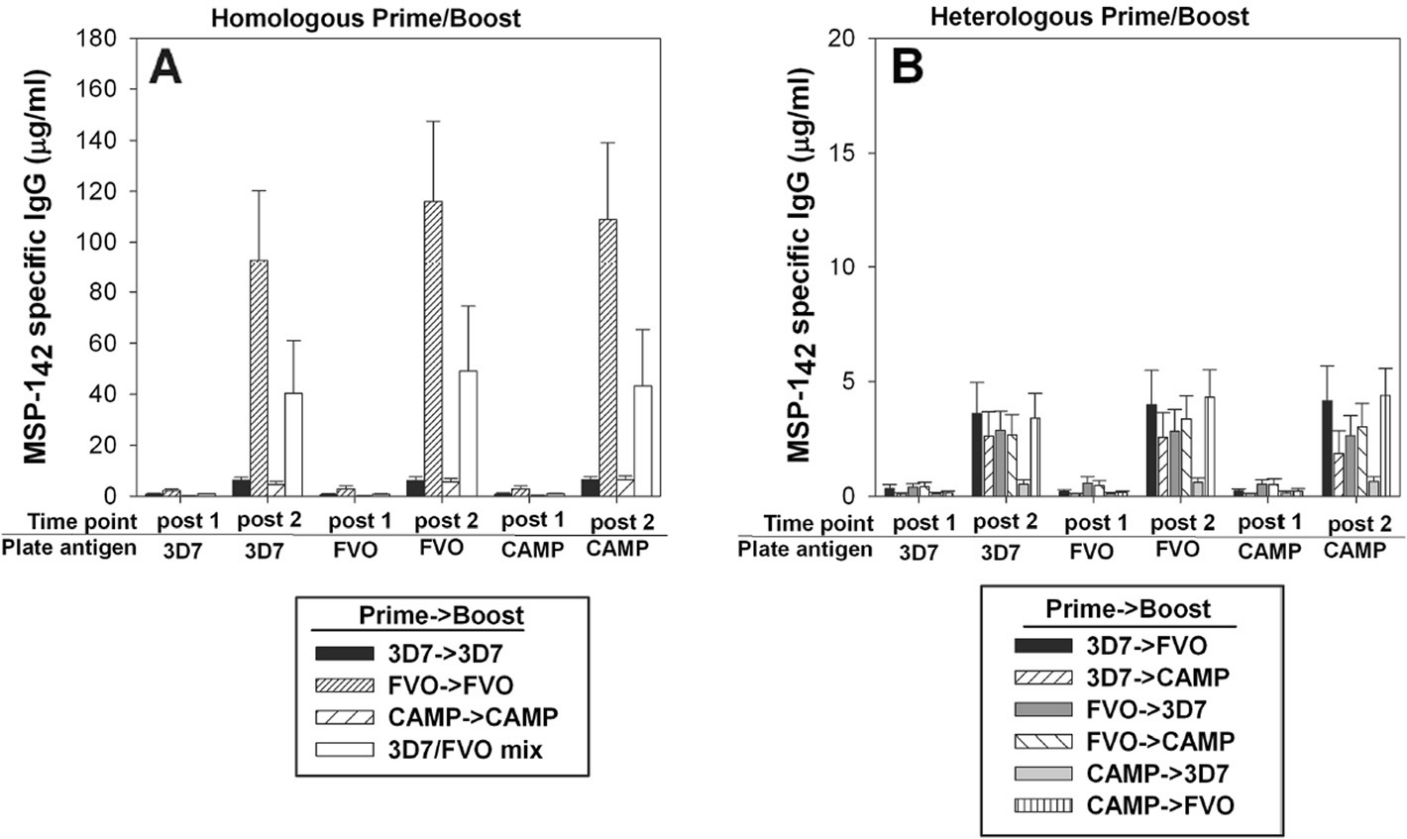
**Differences in the immunogenicity of various MSP1**_**42**_**proteins after homologous and heterologous immunizations of inbred BALB/c were tested by ELISA for reactivity against homologous (Panel A) and heterologous (Panel B) MSP1**_**42**_**proteins.** X-axis = time point (two weeks post first or second immunization) using ELISA plate antigens: MSP1_42_ 3D7, FVO and CAMP. Data are expressed as the mean μg/mL mouse IgG (+/−SEM). n = 20 mice/group, two independent immunization experiments.

**Figure 3:**
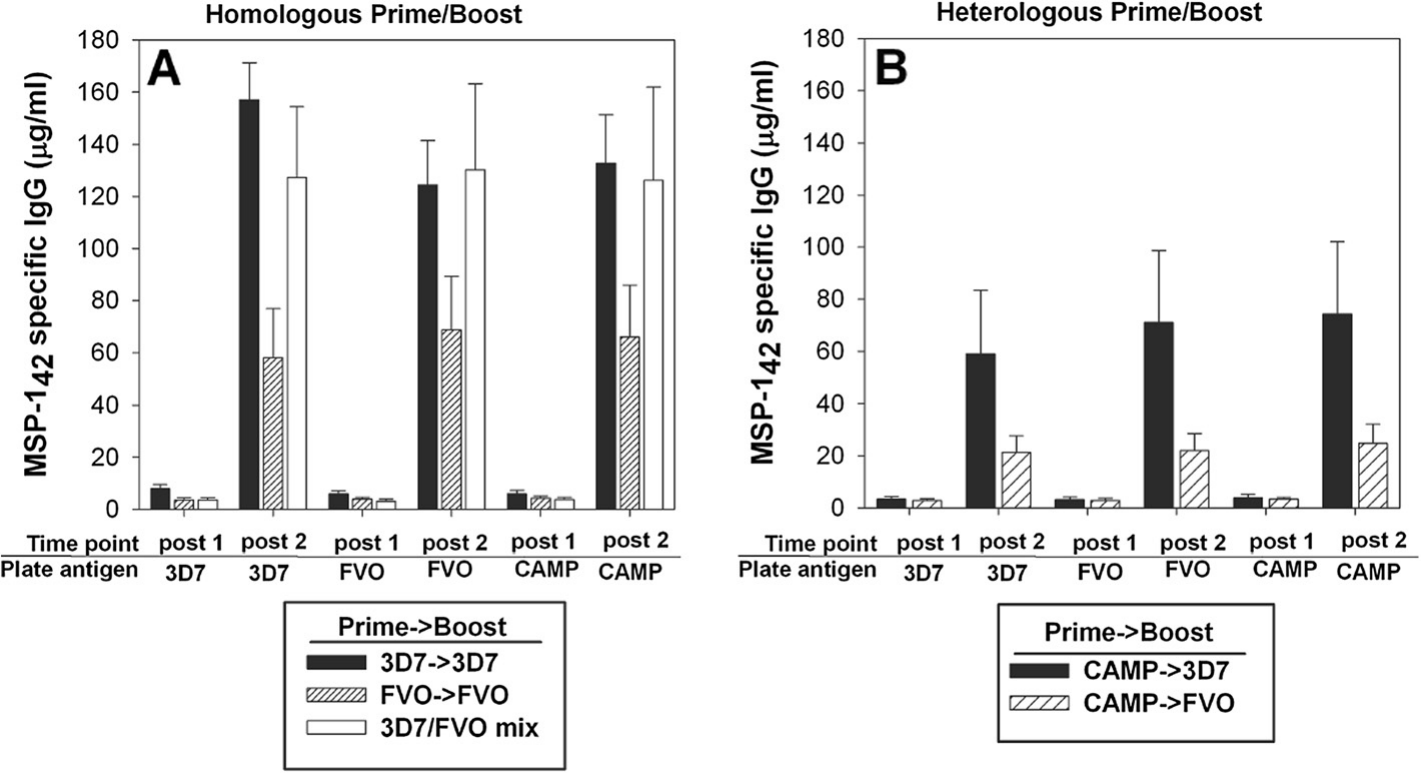
**Differences in the immunogenicity of various MSP1**_**42**_**proteins after homologous and heterologous immunizations of outbred ICR mice were tested by ELISA for reactivity against homologous (Panel A) and heterologous (Panel B) MSP1**_**42**_**proteins.** X-axis = time point (two weeks post first or second immunization) using ELISA plate antigens: MSP1_42_ 3D7, FVO and CAMP. Data are expressed as the mean μg/mL mouse IgG (+/−SEM). n = 20 mice/group, two independent immunization experiments.

MSP1_42_-specific antibody concentrations were measured after the first and the second immunization (Figure 2). In BALB/c mice, the highest antibody concentration was measured after two immunizations with FVO followed by the 3D7/FVO mix (Figure 2A). The difference in antibody concentrations for these two groups were significant only at the 90% confidence level (p = 0.09, 2-sample T-test). These antibodies were equally reactive in the ELISA to both the homologous and the heterologous plate antigens. The two regimens involving identical 3D7 p33 N-termini, 3D7 → 3D7 and CAMP → CAMP, induced relatively poor antibody responses, an order of magnitude lower than for any immunization that involved an FVO immunogen in BALB/c. All heterologous prime and boost regimen induced equally low antibody concentrations after two immunizations suggesting lack of boosting by the second allele (Figure 2B). In ICR mice, the highest antibody responses were induced by the 3D7 → 3D7 and the 3D7/FVO mix (Figure 3). These responses were equally reactive against homologous and heterologous plate antigens (representing the different alleles of MSP1_42_) by ELISA. In regards to the two heterologous prime and boost regimens evaluated in ICR mice (Figure 3), only the mice that were boosted with an immunogen having an identical p33 to the prime (i.e. CAMP → 3D7) induced significant levels of antibodies. These antibodies were equally cross-reactive against all plate antigens (representing the different alleles of MSP1_42_). A disparity in the magnitude of responses induced by the different MSP1_42_ alleles appears mouse strain-dependent and indicates a genetic restriction where BALB/c preferentially recognize the FVO immunogen over the 3D7 and CAMP MSP1_42_ proteins (Figure 2A). This finding emphasizes the necessity to evaluate vaccine combinations in more than one mouse strain.

### Effect of pre-existing immunity on antibody recall responses against homologous or heterologous MSP1 antigens

**Figure 4:**
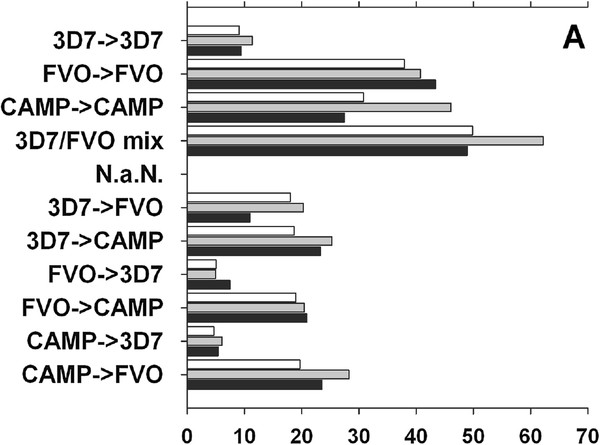
**MSP1-alleles vary in their ability to boost homologous or heterologous MSP1**_**42**_**-induced Ab responses in inbred BALB/c mice (Panel A) and outbred ICR mice (Panel B).** Data expressed as fold increase in antibody concentration of the post 2 over post 1 IgG concentrations. Bar shadings indicate the three MSP1_42_ plate antigens used in the ELISA, No fill (CAMP), gray (FVO), and black filled (3D7).

The data presented in Figures 2 and 3 were further analysed arithmetically to determine the effect of pre-existing immunity on the potency of a heterologous MSP1 allele-based vaccine (Figure 4 A, B). To this end, a stimulation index (SI) was calculated to determine changes in the MSP1-specific antibody levels after the booster immunization. As expected, the homologous cohorts have overall higher indices against all plate antigens compared to the heterologous cohorts except for 3D7 → 3D7 in BALB/c (Figure 4A). The homologous regimen, 3D7 → 3D7, is the weakest of all homologous regimens suggesting a relatively low immunogenicity of this MSP1 vaccine in both mouse strains. Moreover, the 3D7 based vaccine was also inferior when used as the booster immunization compared to the two other allele antigens. In ICR mice, only the ad hoc combination regimen, 3D7/FVO mix, significantly boosts primary responses (Figure 4B). The heterologous regimen CAMP → 3D7 had a much higher boosting index compared to the mis-matched p33 in the CAMP → FVO regimen indicating again the influence of the p33 portion of the molecule to boost pre-existing responses.

### Heterologous immunization regimens affect the epitope fine specificity of humoral responses

**Figure 5:**
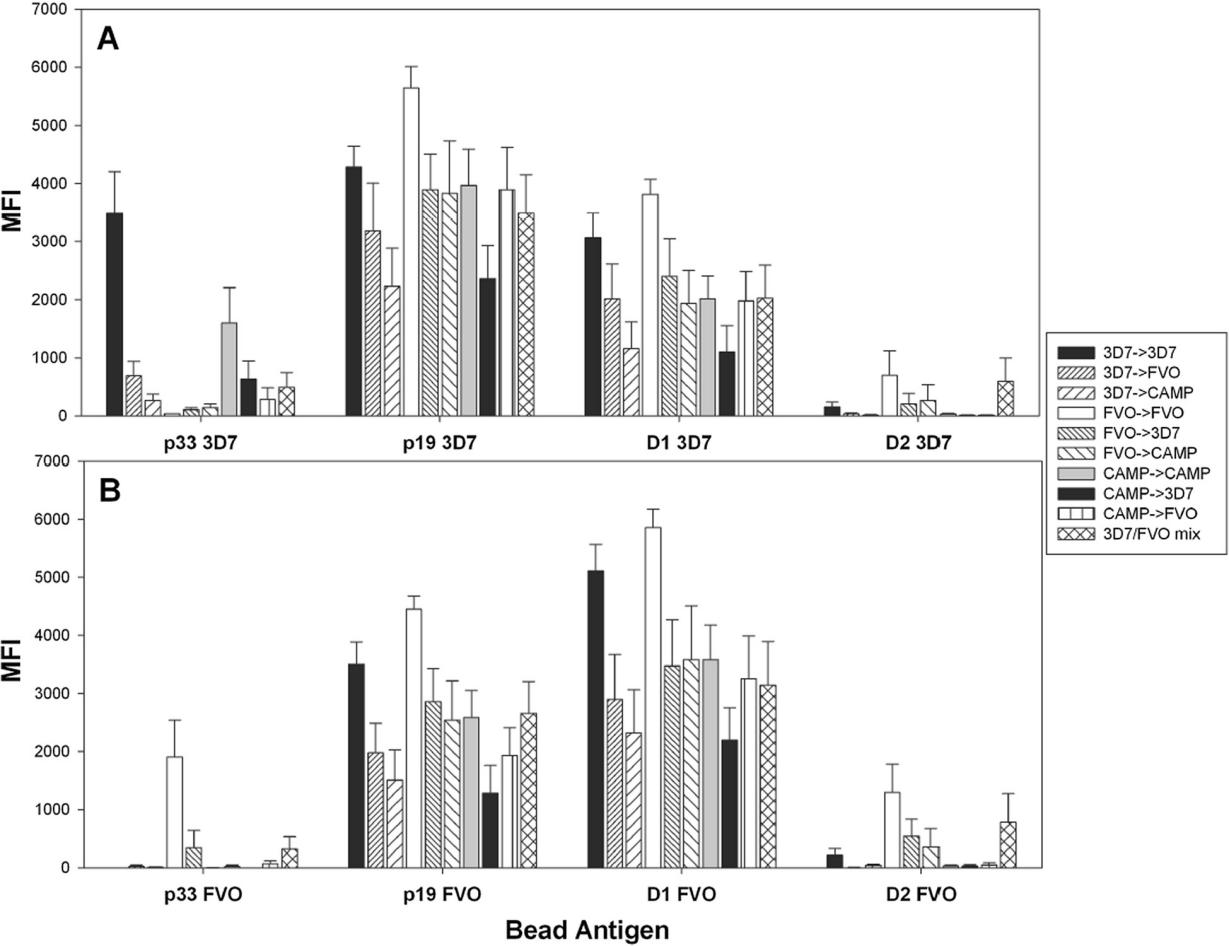
**Heterologous immunization regimens affect the epitope fine specificity of humoral responses in BALB/c mice.** Fine specificities of the post boost sera were tested for reactivity against 3D7 (**Panel A**) and FVO (**Panel B**) protein fragments and analysed by particle-based flow cytometry (Luminex^TM^). Proteins coupled to the Luminex beads are shown on the X-axis. Y-axis = mean fluorescence intensity (MFI). Data are from two independent immunization experiments (n = 20/group).

**Figure 6:**
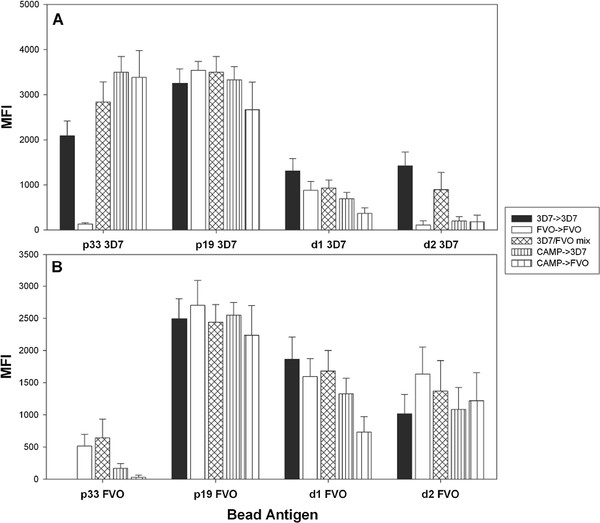
**Heterologous immunization regimens affect the epitope fine specificity of humoral responses in ICR mice.** Fine specificities of the post boost sera were tested for reactivity against 3D7 (**Panel A**) and FVO (**Panel B**) protein fragments and analysed by particle-based flow cytometry (Luminex^TM^). Proteins coupled to the Luminex beads are shown on the X-axis. Y-axis = mean fluorescence intensity (MFI). Data are from two independent immunization experiments (n = 20/group).

To further explore the impact of exposure to different MSP1 alleles on the resulting antibody response the antibody fine specificity of immune sera from BALB/c (Figure 5) and ICR mice (Figure 6) was determined by bead-based flow cytometry (Luminex™). For this purpose, beads were coupled with MSP1_42_ subunit fragments representing the p33, p19 and the EGF-like D1 and D2 for the 3D7 and FVO alleles. Significant allele-specificity was detected to the p33 3D7 (p<0.001, ANOVA) with the highest responses induced by the two p33 homologous regimens (3D7 → 3D7 and CAMP → CAMP). For p19 3D7, the strongest responses were found in the homologous regimen, FVO → FVO, followed by 3D7 → 3D7, and then all regimens that included at least one FVO. Responses to p19 3D7 that were induced by the heterologous EGF-like domain 2 regimens, 3D7 → CAMP and CAMP → 3D7, were the lowest (p = 0.034, ANOVA). A similar trend was seen for p19 FVO responses (Figure 5B) with 3D7 → CAMP and CAMP → 3D7 inducing the lowest responses to both p19 FVO and D1 FVO and all responses to EGF-like D1 paralleled the responses to the p19. These results indicate that the FVO vaccine is more potent than the other MSP-vaccines, as it induces stronger antibody responses even against the heterologous p19 3D7. Immunization with the FVO → FVO and the 3D7/FVO mix regimen led to pronounced responses to the EGF-like domain 2 (D2) of p19 3D7 and FVO. Overall, the FVO → FVO vaccination regimen induced the highest responses to the C-terminus of MSP1_42_.

The fine specificity of immune sera from ICR mice (Figure 6) were noticeably different from those observed for BALB/c mice. Responses to p33 3D7 were relatively high for all regimens tested except for the FVO → FVO (p<0.0001, ANOVA) which failed to induce any cross reactivity to p33 3D7. Responses to p33 FVO were only induced by the two regimens containing homologous p33 FVO in the prime and boost. These findings further underscore the influence of strain specificity at the N-terminal p33 portion of MSP1_42_. None of the regimens could be distinguished based on their responses to p19 3D7 and p19 FVO. On the other hand, a hierarchy of responses was detected to D1 of both p19 alleles with the highest response in the 3D7 → 3D7 and the lowest in the CAMP → FVO regimen (p = 0.018, ANOVA). Induction of specific antibodies to D2 of the p19 3D7 required immunization with either the homologous 3D7 vaccine (3D7 → 3D7) or the homologous 3D7/FVO mix vaccine while the responses tested against the D2 of FVO were overall not distinguishable by vaccination regimen.

### Functional activity of antibodies and the magnitude of the humoral immune response do not parallel each other

**Figure 7:**
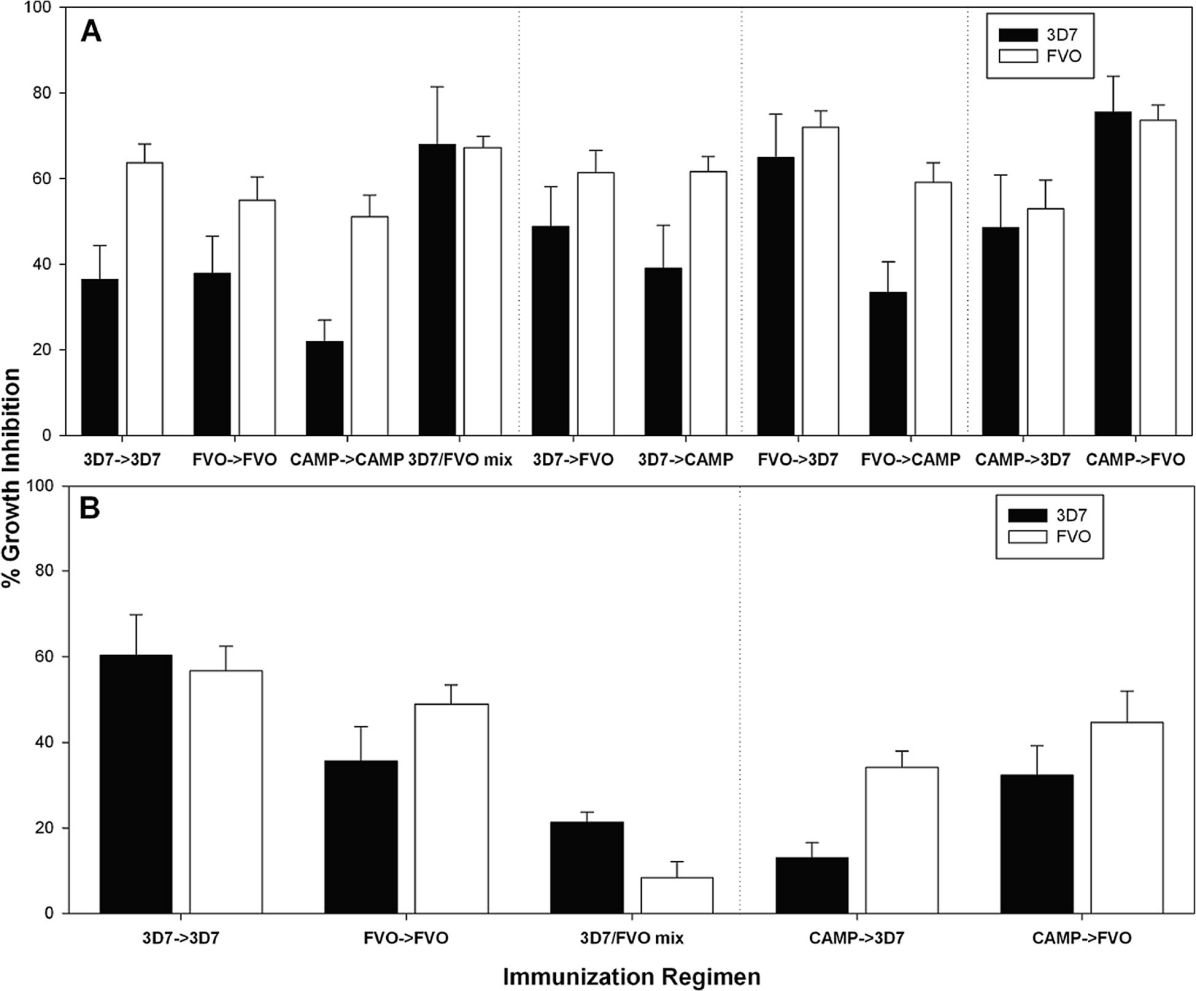
**Functional activity of antibodies induced by immunization does not parallel the magnitude of the serological response.** Growth inhibition of 3D7 (black bars) and FVO (white bars) parasites by MSP1 specific antibodies induced in BALB/c (**Panel A**) or ICR mice (**Panel B**) by the various immunization regimens (indicated on X-axis). Bars represent mean GIA activity (SEM) of 20 individual mice (two independent immunization experiments). Purified immunoglobulins were tested at a final concentration of 0.8 mg/mL.

Previous studies have demonstrated that the quality, and not necessarily the quantity of the MSP1-specific antibody response determine the degree of protection [13,43]. To characterize the quality of the humoral immune response, functional assays were performed with the MSP1-specific antibodies induced by the various immunization regimens. Immunoglobulins from individual mice were tested for anti-parasite activity using the pLDH GIA [19,43]. Analysing the functional activity of antibodies induced in BALB/c mice showed statistically different responses against 3D7 and FVO parasites (p = 0.003 and p = 0.007, respectively, ANOVA) (Figure 7A). Regimens leading to the highest level of growth inhibition against both 3D7 and FVO parasites were the 3D7/FVO mix, CAMP → FVO, FVO → 3D7, and CAMP → 3D7, indicating cross-reactivity at the level of functional antibodies that is not necessarily evident when solely measuring total antibody.

Analysing the functional antibodies induced in ICR mice revealed a significant difference in the level of growth inhibition against both 3D7 and FVO parasites by regimen (p = 0.001 and p<0.001, respectively, ANOVA) (Figure 7B). The 3D7/FVO mix induced relatively low levels of activity against 3D7 parasites and failed to induce significant activity against FVO parasites. Immunization with the 3D7 → 3D7 regimen resulted in the strongest growth inhibition with no discernible preference for the homologous strain. All other regimens induced responses that were higher against the FVO parasites (Figure 7B).

### Cellular immune responses induced by the various regimens are strain-specific

**Figure 8:**
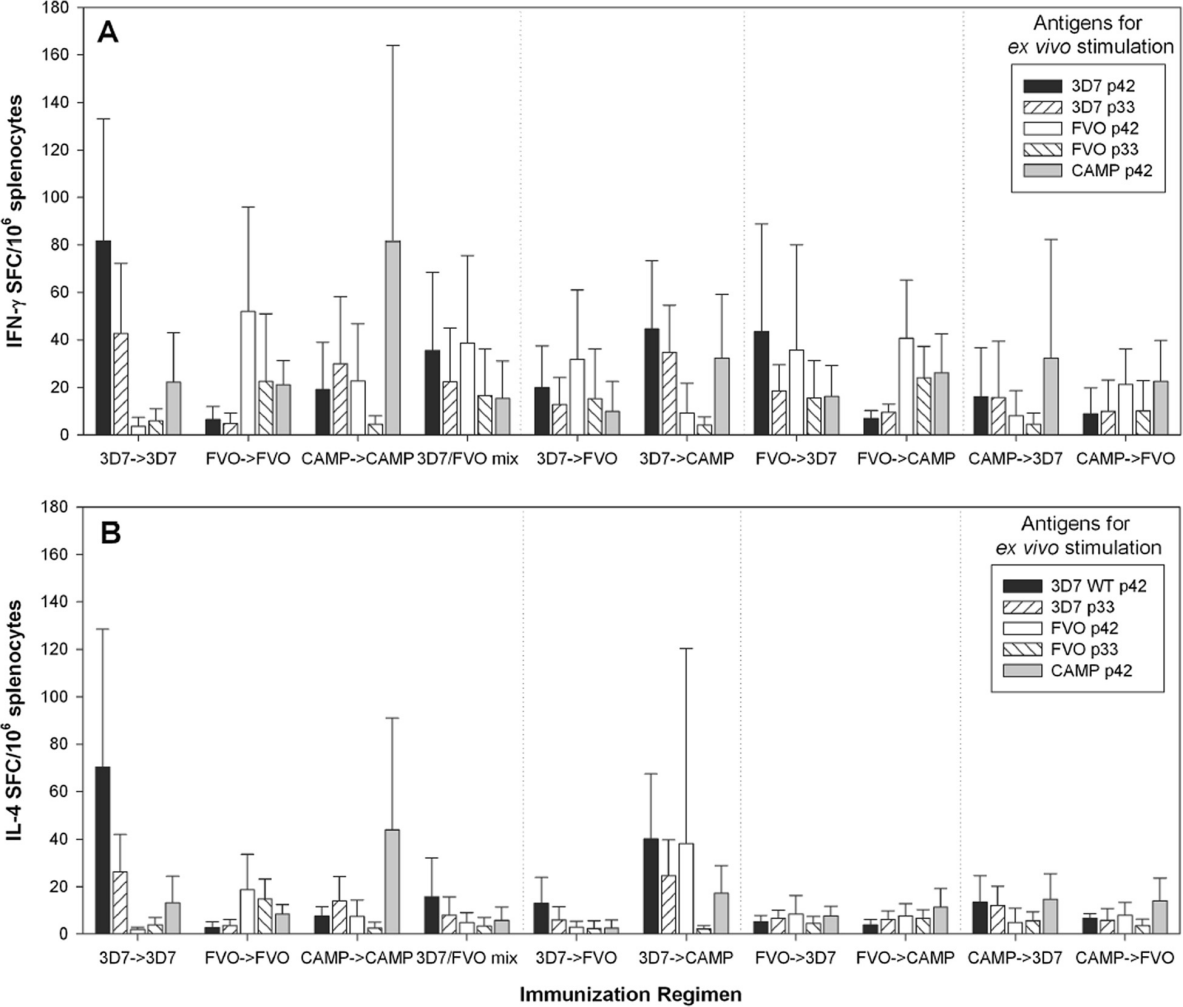
**Cellular immune responses induced by the various regimens are strain-specific.** Fine specificity of T cell responses induced in BALB/c mice (**Panel A**, **B**) by various immunization regimens. IFN-γ responses (**Panel A**) and IL-4 (**Panel B**) responses were measured after *ex vivo* stimulations with MSP1_42_ and subunit fragments (antigen dose for *ex vivo* stimulation = 5 μg/mL) by ELISpot analysis. Data are expressed as the mean (+/− SEM) number of cytokine spot producing cells (SFC) per 10^6^ splenocytes. Data are from two independent immunization experiments (n = 20/group). Cells from mice immunized only with saline/Montanide ISA-720 and splenocytes from the various immunization regimens stimulated with GST as control antigen did not yield responses above medium control.

**Figure 9:**
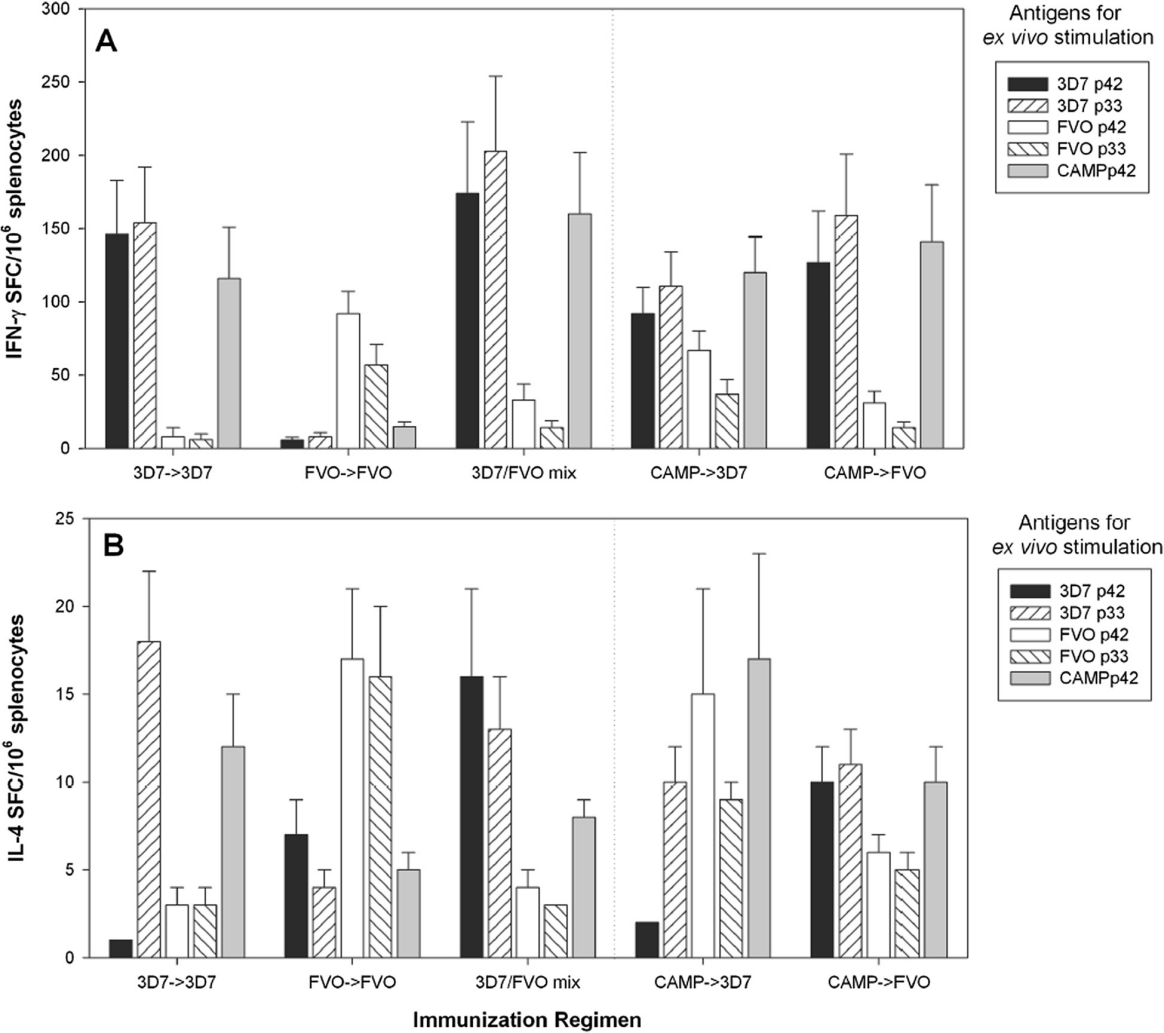
**Cellular immune responses induced by the various regimens are strain-specific.** Fine specificity of T cell responses induced in ICR mice (**Panel A**, **B**) by various immunization regimens. IFN-γ responses (**Panel A**) and IL-4 (**Panel B**) responses were measured after *ex vivo* stimulations with MSP1_42_ and subunit fragments (antigen dose. for *ex vivo* stimulation = 5 μg/mL) by ELISpot analysis. Data are expressed as the mean (+/− SEM) number of cytokine spot producing cells (SFC) per 10^6^ splenocytes. Data are from two independent immunization experiments (n = 20/group). Cells from mice immunized only with saline/Montanide ISA-720 and splenocytes from the various immunization regimens stimulated with GST as control antigen did not yield responses above medium control.

In order to determine the fine specificity of the cellular immune response, the number of antigen-specific splenic T cells secreting either IFN-γ (Figures 8A and 9A) or IL-4 (Figures 8B and 9B) was determined *ex vivo* by ELISpot analysis. Initially, a comprehensive analysis was performed on splenocytes from BALB/c mice obtained from all regimens tested. Splenocytes were stimulated with recombinant MSP1_42_ CAMP, 3D7 and FVO, p33, p19 and each of the subunit fragments for D1 and D2 of both 3D7 and FVO clones (Figure 8 and Table 1). Since no MSP1-specific responses against the smaller subunits p19, D1 and D2, above background levels were detected (stimulation above glutathione S-transferase (GST) alone), these data were omitted from further analyses. In contrast, significant responses were measured against the larger subunit fragments MSP1_42_ and the p33. The highest IFN-γ responses were measured in the 3D7 → 3D7 and CAMP → CAMP regimens and to a lesser extent in the 3D7 → CAMP and FVO → 3D7 after *ex vivo* stimulation with MSP1_42_ 3D7 and p33 3D7 (p = 0.0007 and p = 0.0176, respectively, two-sided T-test). For FVO stimulations with either MSP1_42_ or p33, the IFN-γ responses did not significantly differ (p = 0.148 for the MSP1_42_ FVO and p = 0.299 for the p33 FVO). Regimens that included a least one FVO immunization were more likely to recall FVO specific responses. The highest degree of strain-specificity was observed with the CAMP → CAMP regimen as this group responded stronger to the *ex vivo* stimulation with the MSP1_42_ CAMP than to any other antigen (p = 0.043, two-sided T-test). The IL-4 responses, although lower overall, paralleled the IFN-γ responses: The highest IL-4 responses were measured after stimulation with MSP1_42_ 3D7 and p33 3D7 in the 3D7 → 3D7 and CAMP → CAMP regimens (p = 0.0002 and p = 0.0001, respectively, two-sided T-test). Only the 3D7 → CAMP regimen induced responses that could be significantly stimulated by MSP1_42_ FVO stimulation.

**Table 1:** Reactivity of MSP1 specific T cells from BALB/c mice demonstrates strong strain-specificity

**Regimen**	**IFN-γ responses**^**a**^			**Il-4 responses**^**a**^		
	**MSP1**_**42**_			**MSP1**_**42**_		
	**CAMP**^**b**^	**FVO**	**3D7**	**CAMP**	**FVO**	**3D7**
FVO → FVO	+	**+++**	+	+/−	**+**	+/−
FVO → 3D7	+	++	++	+/−	+/−	+/−
FVO → CAMP	+	++	+/−	+	+/−	+/−
3D7 → 3D7	+	+/−	**++**	+	+/−	**+++**
3D7 → FVO	+/−	++	+	+/−	+/−	+
3D7 → CAMP	++	+/−	++	+	++	++
3D7/FVO mix	+	++	++	+/−	+/−	+
CAMP → CAMP	**+++**	+	+	**++**	+/−	+/−
CAMP → 3D7	++	+/−	+	+	+/−	
CAMP → FVO	+	+	+/−	+	+/−	+/−

^a^ Strength of response: + ≥ 15 SFC, ++ ≥30, +++ ≥ 45 SFC. Responses of mice immunized with saline/Montanide ISA-720 did not exceed 2 ± 1 SFC/10^6^ splenocytes when stimulated with any of the antigens used. Responses of splenocytes stimulated with GST as the control antigen did not exceed 2 ± 2 SFC/10^6^ splenocytes.

^b^ MSP1_42_ allele used for *ex vivo* stimulation. Bold characters highlight the magnitude of the homologous response.

A select set of immunization regimens (-exposure in Western Kenya where CAMP parasites predominant and either a 3D7 or FVO allele vaccine could be deployed) were employed to investigate the fine specificity of T cell responses in outbred mice (Figure 9 and Table 2). Similar to the findings for inbred BALB/c mice, the IFN-γ responses in ICR mice demonstrated a strain-specific response pattern. The regimens that included the 3D7 vaccine yielded higher T cell responses to MSP1_42_ 3D7 and p33 3D7. The regimens that included the CAMP or FVO vaccine induced higher responses to MSP1_42_ FVO and p33 FVO (Figure 9A). The IL-4 responses induced by the various regimens paralleled the T cell fine specificity seen for the IFN-γ responses (Figure 9B). As expected, IL-4 responses induced in ICR mice were lower than the responses measured in BALB/c mice as the latter strain of mice is biased towards Th2-type responses [44].

**Table 2:** Reactivity of MSP1 specific T cells from ICR mice demonstrates strong strain-specific IFN-γ responses

**Regimen**	**IFN-γ responses**^**a**^			**Il-4 responses**^**a**^		
	**MSP1**_**42**_			**MSP1**_**42**_		
	**CAMP**^**b**^	**FVO**	**3D7**	**CAMP**	**FVO**	**3D7**
FVO → FVO	+/−	**+++**	+/−	+/−	**+**	+/−
3D7 → 3D7	+++	+/−	**+++**	+/−	+/−	**+/−**
3D7/FVO mix	+++	+++	+++	+/−	+/−	+
CAMP → 3D7	+++	+++	+++	+/−	+/−	+/−
CAMP → FVO	+++	+/−	+++	+/−	+/−	+/−

^a^ Strength of response: + ≥ 15 SFC, ++ ≥30, +++ ≥ 45 SFC. Responses of mice immunized only with saline/Montanide ISA-720 did not exceed 3 ± 2 SFC/10^6^ splenocytes when stimulated with any of the antigens used. Splenocytes stimulated with GST as control antigen did not exceed 2 ± 2 SFC/10^6^ splenocytes.

^b^ MSP1_42_ allele used for *ex vivo* stimulation. Bold characters highlight the magnitude of the homologous response.

## Discussion

Evidence of protection induced by MSP1_42_ based vaccines in nonhuman primate studies [11,13,34] and the association of MSP1 antibodies with reduced parasite burden and clinical disease (reviewed in [45,46]) have contributed to the development of recombinant MSP1-based vaccine candidates for clinical investigations. However, significant obstacles to overcome for blood stage vaccines that have yet to be addressed include the issues of parasite strain heterogeneity in the field and pre-existing immunity to circulating alleles. The present study models the impact of an established immune response to one MSP1_42_ allele (prime) on the induction of immunity to a different allele (boost). To this end, recombinant MSP1_42_ protein antigens from the two major MSP1_42_ alleles, namely MAD20 (3D7 clone) and Wellcome/K1 (FVO clone) and the CAMP clone, a recombinant between MAD20 and Wellcome/K1 (Figure 1) adjuvanted with Montanide ISA-720 were used as immunogens in various prime:boost regimens.

Analysis of antibody responses revealed that in the context of homologous immunization, both the FVO → FVO and the 3D7/FVO mix were highly immunogenic in BALB/c (Figure 2). These antibodies had a high degree of cross-reactivity to each of the *P. falciparum* strain MSP1_42_ antigens by ELISA, suggesting that these two regimens could overcome the allelic diversity in the field. In addition, the BALB/c mice model yielded reduced immunogenicity of the homologous 3D7 and CAMP vaccines, most likely due to a genetic restriction within this mouse strain. Humoral responses in ICR mice were higher when immunizing with the 3D7 vaccine, in contrast to the responses seen in BALB/c mice.

A stimulation index (SI) was calculated to assess the effect of pre-existing immunity on the ability of a homologous or heterologous vaccine to boost an established humoral immune response (Figure 4A, B). The SI reports the fold increase of MSP1_42_–specific antibody concentration between the prime and booster immunization. In all cases, a homologous prime:boost out performed any heterologous regimens in terms of the ability to boost pre-existing immunity in BALB/c mice. In ICR mice, none of the regimens performed as well as the 3D7/FVO mix. These data reveal that in an experimental murine model, the priming exposure to MSP1-alleles leads to “clonal imprinting” also known as original antigenic sin. For *Plasmodium*, this is the first such demonstration of clonal imprinting and these findings have major implications for the development of blood stage malaria vaccines. Similar evidence of pre-exposure has previously been shown to modulate subsequent immune responses (reviewed in [32,33]).

Analysis of the antibody fine specificity by Luminex™ induced in inbred and outbred mice by the various regimens provided several important insights: (1) Responses to the p33 3D7 were obtained only when using immunization regimens that consisted of either homologous CAMP or 3D7 regimens, or any regimens that included the 3D7 vaccine. Responses in BALB/c mice to the p33 FVO were only significant in the homologous FVO → FVO regimen. In ICR mice, p33 FVO responses were only induced by the homologous FVO → FVO regimen and the 3D7/FVO mix regimen. These results clearly show that the responses to p33 are not cross-reactive between the alleles: no boosting occurs in the presence of an established heterologous allele response. (2) Anti-p19 responses did not show any allele specific immune responses. (3) Responses to the D2 of p19 3D7 and FVO were only triggered by the 3D7/FVO mix regimen and the FVO → FVO regimen in BALB/c mice. In ICR mice, the 3D7/FVO mix regimen and the 3D7 → 3D7 regimen were able to induce responses to the D2 of the p19 3D7 while any regimen tested was able to induce responses to the D2 of the p19 FVO.

The most stringent measurement of a vaccine’s effectiveness is to determine the biological effect of the induced immune response. Therefore, the growth inhibitory activity of antibodies generated by the various regimens was determined (Figure 7). The regimen that induced antibodies with the highest anti-parasite activity against both parasite strains in BALB/c mice was CAMP → FVO. In ICR mice, the strongest functional responses were induced by the 3D7 → 3D7 regimen whereas the 3D7/FVO mix vaccine triggered the weakest functional antibody response, a result inconsistent with the response seen in BALB/c mice. These GIA data suggest that the high titer antibodies induced by the 3D7/FVO mix may be to epitopes that do not necessarily function against the parasite. It should, however, be noted, that although routinely used as a surrogate readout for vaccine efficacy, there is no evidence that the *in vitro* growth-inhibitory activity correlates to biological relevance of antibodies against blood stage antigens [46]. While MSP1_19_-specific GIA active antibodies acquired through natural immunity have been associated with reduced clinical disease [7,47], confounding evidence suggests that the quality of antibodies induced in malaria-naïve- and malaria-experienced individuals vaccinated with blood stage antigens differs [48-51]. It is not clear whether the inability of blood stage vaccines to induce functional antibody responses to MSP1 in the field is solely due to the complexity and diversity of circulating parasite strains [24,52,53] or to the ability of the vaccine to overwrite pre-existing immunity. These findings suggest that assessing a vaccine-induced growth inhibitory activity, in the context of natural immunity, is hindered by the multitude of antibody specificities.

Induction of potent humoral immune responses requires the engagement of an efficacious T helper cell response. Evidence in the literature suggests a direct involvement of effector T cells in protection against blood stage malaria [54]. Analysing the fine specificity of the T cell response revealed that the homologous regimens were highly immunogenic for inducing IFN-γ responses in BALB/c mice. In contrast, the 3D7 and CAMP vaccines were more immunogenic in ICR mice. These results confirm the existence of an MHC-restriction in BALB/c that favors the presentation of FVO-derived peptide fragments over 3D7 derived fragments. When using the full length *vs* fragments of MSP1 to stimulate recall responses, only the full length and the N-terminal p33 fragments were able to stimulate T-cell responses *ex vivo*. In part, this may be a result of the disulfide constrained nature of the p19, which can impede antigen processing [55]. Cellular responses revealed a p33-dominated response in the regimens where the vaccines shared the same p33 amino acid sequence (3D7 and CAMP) leading to a boost of the cellular response. In no case did a heterologous p33 regimen induce cross-reactivity. The current study reveals that despite an allele-specific T cell response; an allele-cross-reactive humoral immune response could be induced leading to antibodies that recognize parasite clones with homologous and heterologous MSP1 alleles.

Natural immunity to malaria is an age-dependent phenomenon developing over repeated infections. The immunity that develops becomes broadly specific and comprises antibodies induced to the blood stages of infection. Initially, the immunity that develops is allele-specific, and thus is inadequate to protect against heterologous exposure. To date, the immunity induced by blood stage vaccines is allele-specific, highlighted in several Phase 2b field trials where vaccines representing single alleles were evaluated to MSP2 in Combination B (Papua New Guinea) [48], AMA1 3D7 in FMP2.1/AS02_A_ (Mali) [53,56], and MSP1_42_ 3D7 in FMP1/AS02_A_ (Western Kenya) (personal communication C.F. Ockenhouse). From these studies, it is reasonable to conclude that multiple-allelic formulations are required to overcome the effect of allelic polymorphisms on induced immune responses. However, in the context of the current study, none of the regimens that were tested were able to circumvent allele-specific immunodominant T cell responses. Overcoming this limitation may require frequent, sequential prime and boost regimens with heterologous immunogens focusing immune responses toward more broadly conserved and cross reactive epitopes. The current model of pre-existing immunity did not adequately address this question since mice were only immunized two times. Additional studies will be required to determine whether serial immunizations with heterologous immunogens can overcome the allele specificity at the T cell level. Thus, in the context of the current study, immunizing infants and young children (without previous malaria exposure) with the two dimorphic alleles of MSP1_42_ should suffice to prime for conserved cross-reactive responses that are directed against parasites that they may encounter later. While in adult vaccinees, their extensive exposure and pre-existing immunity should allow recognition of conserved epitopes on vaccine immunogens leading to successful boosting of these responses through repeated exposure.

## Conclusion

The present study demonstrates that pre-existing immunity modulates the magnitude and specificity of immune responses induced by subsequent immunizations. The effect was pronounced at the level of T cell responses. The degree of homology in the p33 region of MSP1 between the allele responsible for the clonally imprinted immune response and the vaccine allele determines the magnitude of vaccine induced responses.

## Competing interests

EA holds several patents on MSP1 protein vaccines.

## Authors’ contributions

EBL co-designed the study, drafted the manuscript, conducted growth inhibition assays and ELISpot assays and did the statistical analysis. ED and RM conducted the serological characterization and ED edited the manuscript. EA co-designed the study, directed the work and edited the manuscript. All authors read and approved the final manuscript.

## Disclaimer

The authors’ views are private and are not to be construed as official policy of the Department of Defense or the U.S. Army. Research was conducted in compliance with the Animal Welfare Act and other federal statutes and regulations relating to animals and experiments involving animals and adheres to principles stated in the *Guide for the Care and Use of Laboratory Animals*, NRC Publication, 1996 edition.
